# Development of a new and facile method for determination of chlorpyrifos residues in green tea by dispersive liquid–liquid microextraction

**DOI:** 10.1038/s41598-022-20021-0

**Published:** 2022-09-15

**Authors:** Hai Tian, Yujie Feng, Xinfeng Yang, Shuhuai Li, Chaohai Pang, Chen Ma

**Affiliations:** 1 Analysis and Testing Center, Chinese Academy of Tropical Agricultural Science & Hainan Provincial Key Laboratory of Quality and Safety for Tropical Fruits and Vegetables, Haikou, China; 2 Institute of Plant Protection, Ministry of Agriculture, Hainan Academy of Agricultural Science & Scientific Observation and Experiment Station of Crop Pests in HaiKou, Haikou, China

**Keywords:** Environmental chemistry, Analytical chemistry

## Abstract

In this work a simple, rapid, and environmentally friendly method has been established for the determination of chlorpyrifos residue in green tea by dispersive liquid–liquid microextraction and gas chromatography-flame photometric detection. Some experimental parameters that influence extraction efficiency, such as the kind and volume of disperser solvents and extraction solvents, extraction time, addition of salt and pH, were investigated. And the optimal experimental conditions were obtained, quantitative analysis was carried out using external standard method. The correlation coefficient of the calibration curves was 0.999 with in 0.05 mg/kg to 5 mg/kg. The results showed that under the optimum conditions, the enrichment factors of the chlorpyrifos was about 554.51, the recoveries for standard addition fell in the range from 91.94 to 104.70% and the relative standard deviations was 4.61%. The limit of quantification of chlorpyrifos in green tea was 0.02 μg/mL at the signal/noise ratio of 3.

## Introduction

Tea originated in China and has a history of more than 6000 years. Together with cocoa and coffee, tea is one of the world's top three beverages. Tea is produced by more than 45 countries and consumed by over two-thirds of the world 104.70% and^1^, with China being the biggest tea producer and the second largest exporter in the world^2,3^. There are many kinds of tea, green tea is an unfermented tea produced from fresh leaves of Camellia sinensis plant, green tea is the world's leading tea, accounting for about 50% of the world's total tea output. It has been a popular beverage for many centuries, particularly in East Asian countries, and is becoming increasingly popular worldwide mainly because of its flavour quality^4–6^ and potential health benefits^7–9^.

In the modern agricultural industry, owing to the occurrence of pests, diseases, and weeds, it is unavoidable to use plenty of pesticides (e.g. insecticides, fungicides, and herbicides) during the growth of crops to ensure high production and quality^10^. During the period of tea growth and harvest, various of pesticides are applied to prevent injurious insect to guarantee the production and cultivation^11,12^. Chlorpyrifos is a kind of broad spectrum, efficient, moderate toxicity, and long residual effect period of organophosphorus pesticides, has a good stomach toxicity and contact action, it is mainly used for the prevention and treatment of cotton, vegetables, tea, fruit and crops on the harmful insects and mites^13–15^. However, the extensive use of chlorpyrifos in agriculture leaves chemical residues on food commodities, including tea leaves. This serves as source of chlorpyrifos residues in tea for consumers^16^. Although pesticides play a significant role in increasing crop production, high levels of residual pesticides can result in adverse effects on the food safety and human health^17^. In recent years, with the increasing concern on the health risks caused by pesticide residues, for the toxicity of chlorpyrifos has already had a relevant research^18–21^, the safety of tea is receiving greater attention by both consumers and industry players. To ensure consumers' health and safety, many countries and international organizations have established maximum residue levels (MRLs) for chlorpyrifos in tea. The MRLs of chlorpyrifos in tea set by European Union, United States, Japan and China are 0.01 mg/kg, 0.1 mg/kg, 10 mg/kg and 2 mg/kg, respectively.

Detection of pesticide residues is essential in regulating and monitoring the levels of pesticide contamination^16^. Currently. some classical analytical methods such as highperformance liquid chromatography^22^, liquid chromatography-mass spectrometry^23,24^, gas chromatography-mass spectrometry (GC–MS)^25,26^ were used to analyze pesticide residue in tea^11^. But, the factors such as low concentration of the analytes and the presence of different interferences in the matrices of samples limit the direct application of these instruments despite of their high sensitivity^27^. Therefore, it is very important to extract/preconcentrate analytes from the sample matrix for sample pretreatment. Traditional extraction methods for pesticide residues are solid phase extraction (SPE)^27,28^ and liquid–liquid extraction (LLE)^29^. The main difficulties of these methods are the use of large amounts of toxic organic solvents (in LLE), the blocking of the cartridge, and the time consuming (in SPE)^30,31^.

To solve these problems, in 2006, Rezaee etc^32^. first reported the dispersive liquid–liquid microextraction (DLLME) technology. In this technology, a mixture of extraction and dispersive solvents is hastily injected into the aqueous phase of the analyte^33^. By doing so, analytes are extracted into the formed fine droplets of the extraction solvent. This sample pretreatment technology is integrates sampling, extraction and concentration into an organic whole, the extraction solvent is used only at μL-level, with a series of advantages including operation is simple, fast, low cost, high enrichment efficiency and environmentally friendly^34–36^. At present, the DLLME method has been applied to the organophosphorus pesticide^37–43^, carbamates pesticide^42–46^, neonicotinoid^47^, polycyclic aromatic hydrocarbons^48–51^ and other organic pollutants analysis determination.

In this work, for the first time, an efficient sample pretreatment method based on DLLME was established for the extraction of chlorpyrifos residue in green tea, and then quantified by gas chromatography-flame photometric detector (GC-FPD). It should be pointed out, that DLLME technology has been reported for the determination of chlorpyrifos residue in fruits, vegetables and Chinese herbal medicine^52,53^, but the application of this technology in the determination of chlorpyrifos residue in green tea has been rarely reported. The study screened efficient dispersant (acetone) and extracting agent (trichloroethane), by evaluating the salt concentration, extraction time, extraction volume, and so on factor's influence on the extraction effect, such as to establish the best extraction conditions. For the first time, the method was applied to detection of chlorpyrifos in complex matrix sample-green tea, the accuracy and sensitivity of the method satisfies the requirement of pesticide residue analysis. Ease of operation, low-cost, and rapidity can be the main advantages of the proposed method. This method was solved the problem of DLLME appaly to the analysis of complex matrix sample-green tea, and has important research value for the efficient extraction and detection of chlorpyrifos in green tea.

## Materials and methods

### Instruments and reagents

Gas chromatography was an Shimadzu GC-17A (Shimadzu, Japan company), equipped with a FPD detector and an analytical column DB-1701 Ultra Inert capillary column (30 m length × 0.53 mm I.D. × 1 μm film thickness, Agilent Technologies, USA); Pipetting gun 10–100 μL (German Brand); 10 mL glass centrifuge tube plug pointed bottom; 1 μL injection needle (Hamilton, Switzerland company).

Chlorpyrifos (certified analytical standard, 98%) was purchased from Dr. Ehrenstorfer company (Germany); Carbon tetrachloride(CTC), trichloroethane (TCE), chlorobenzene (MCB), acetone (analysis of pure) was purchased from Shanghai chemical reagent co., LTD (Shanghai, China); Acetonitrile (chromatography, American Fesher company); Experiment with water for Milli-Q pure water (Milipore companies in the United States). Green tea to buy in a store.

### Chlorpyrifos solution

The confecting of chlorpyrifos standards: according to samples from 102.0 mg chlorpyrifos standards in 100 mL volumetric flask and dissolved in acetone and constant volume, the mixture of 1000 mg/L standard stock solution; Take the standard stock solution with acetone diluted 10 mg/L chlorpyrifos standard solution.

### GC-FPD analytical condition

**Figure 1:**
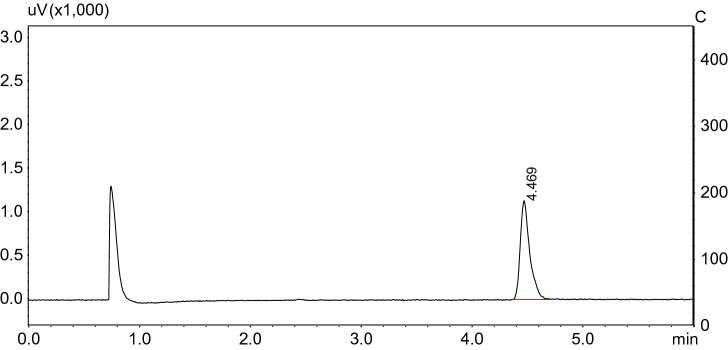
Chromatogram of chlorpyrifos.

Injector temperature, 220 °C, splitless; detector temperature, 250 °C; oven temperature program starting at 170 °C, and ramp 20 °C min^−1^ to 210 °C, 0.5 min at 210 °C, and then ramp 10 °C min^−1^ to 230 °C, 3 min at 230 °C, a total of 7.5 min. Under these conditions chlorpyrifos retention times were approximately 4.4 min (Fig. 1).

### Sample preparation

Take green tea sample 20 mL into 100 mL measuring cylinder, then add 4 g NaCl and 50 mL of acetone solution. thermal agitation after 3 min, room temperature let stand for 30 min, take 1 mL solution (dispersant), to be the next step.

### DLLME procedure

**Figure 2:**
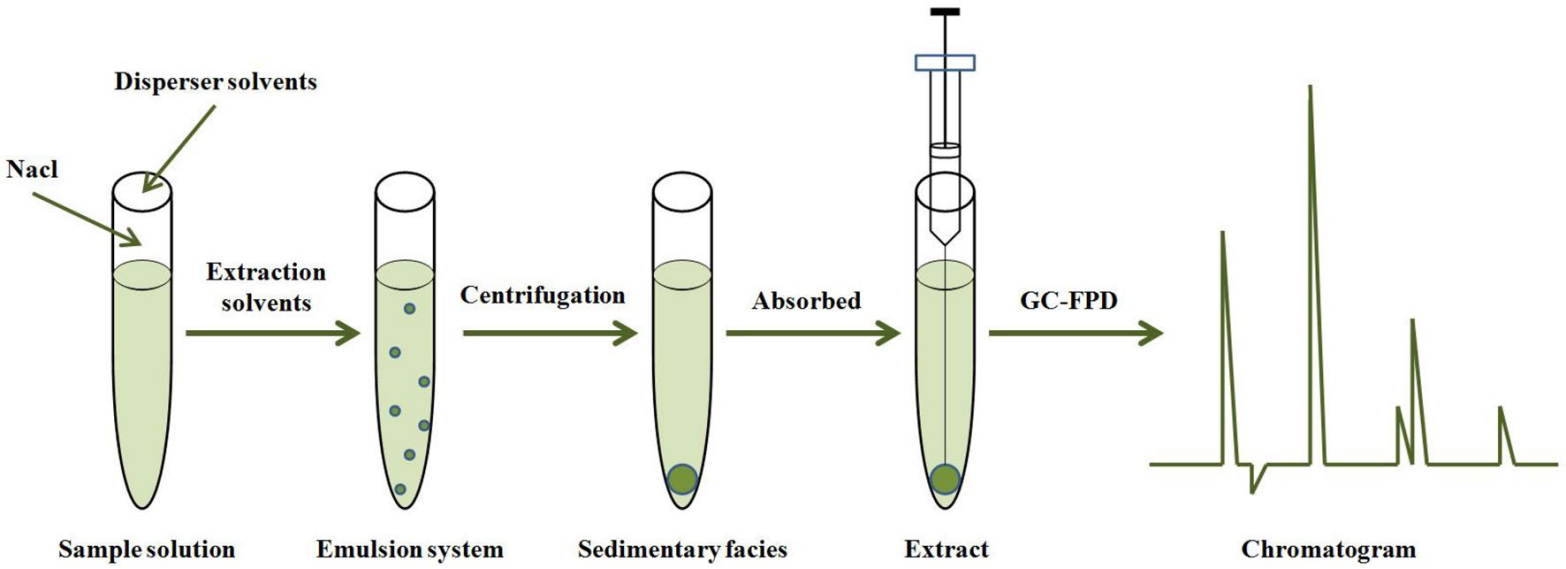
The schematic diagram of the sequential DLLME method.

Take 1 mL dispersant and 22 μL extraction solvent, in turn, add to 10 mL Sharp bottom plug centrifuge tube, gently shake. Then add 5 mL ultrapure water, gently oscillation, extracting agent evenly dispersed in the water phase, the formation of water/dispersant/extraction agent emulsion system, place 2 min at room temperature. Then to 3500 r/min 2 min, the centrifugal extraction agent deposit in the bottom of the centrifuge tube, trace sampler has absorbed 1 μL sedimentary facies, the GC analysis (Fig. 2).

## Results

### Effect of the type and volume of extraction solvent

**Figure 3:**
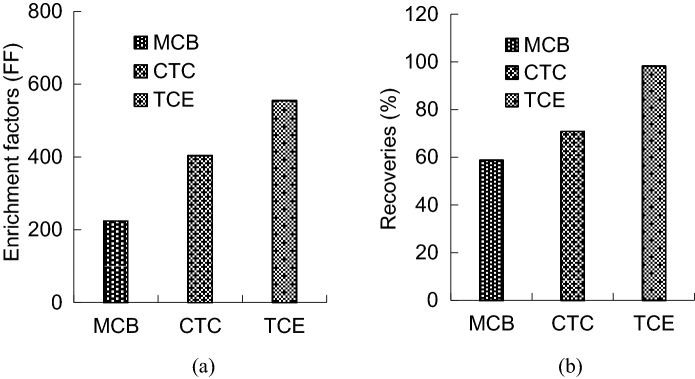
Different type of extraction solvent, (**a**) Enrichment factors of different type of extraction solvent, (**b**) Recoveries of different type of extraction solvent.

This experiment select 3 kinds of organic solvent is chlorobenzene (density of 1.10 g/mL), carbon tetrachloride (density of 1.59 mg/L) and trichloroethane (density of 1.35 g/mL), according to section 1.5 steps, the extraction effect of 3 kinds of extraction solvent on of chlorpyrifos has tested. The results show that (as shown in Fig. 3), trichloroethane has the highest concentration coefficient and good recovery, extraction effect was better than the other two. so choose trichloroethane as extracting agent in this project.

**Figure 4:**
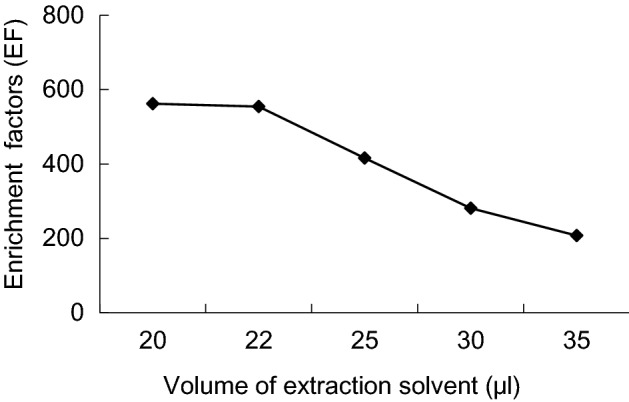
Enrichment factors of different volume of extraction solvent.

**Figure 5:**
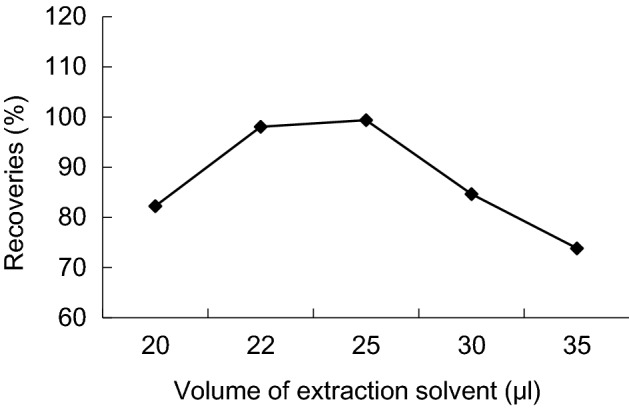
Recoveries of different volume of extraction solvent.

The extraction solvent volume affect the enrichment ratio of DLLME directly, thus affecting recovery, 5 extraction volume have selected in this experiment: 20, 22, 25, 30 and 35 μL. Results shows, that with the increase of the extractant volume, recoveries first increases then decreases by chlorpyrifos, 22, 25 μL are can achieve ideal recovery, but with increase of the extractant volume, the enrichment factor decreases obviously. As 22 μL of the extractant volume, the recoveries and enrichment factor are better (Figs. 4, 5).

### Effect of the type and volume of dispersant solvent

**Figure 6:**
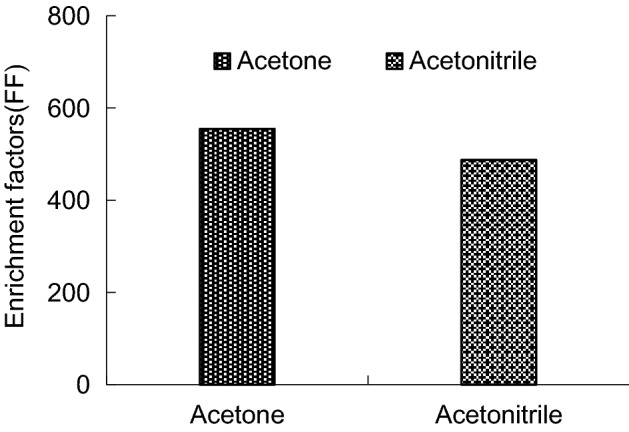
Enrichment factors of different type of dispersant.

**Figure 7:**
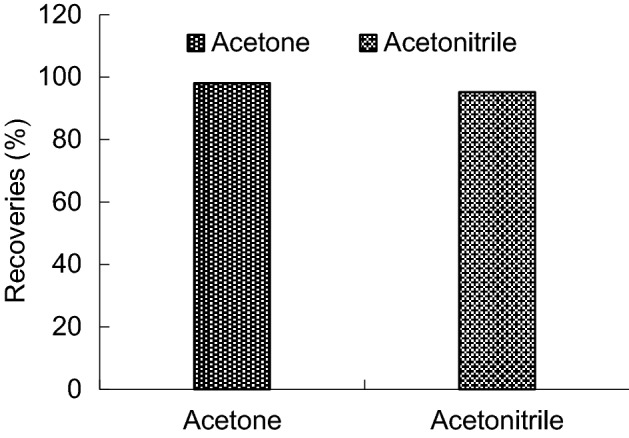
Recoveries of different type of dispersant.

This experiment choose acetonitrile and acetone as dispersant, mix with 22 μL trichloroethane respectively, and add 5 mL water. The acetone extraction efficiency highest, the acetonitrile extraction rate is lower than acetone, and the peak has interference (Figs. 6, 7). So the experiment choose acetone as dispersant.

**Figure 8:**
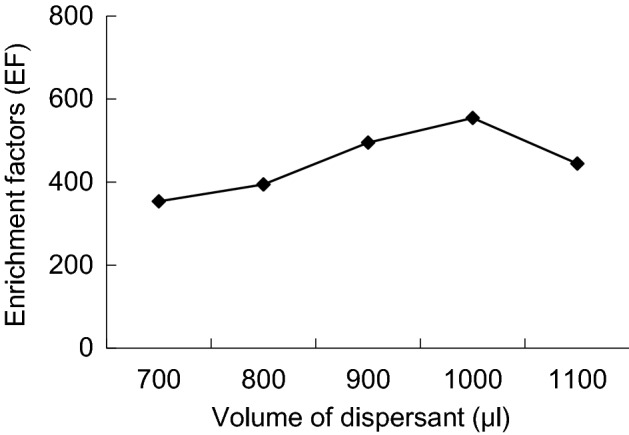
Enrichment factors of different volume of dispersant.

**Figure 9:**
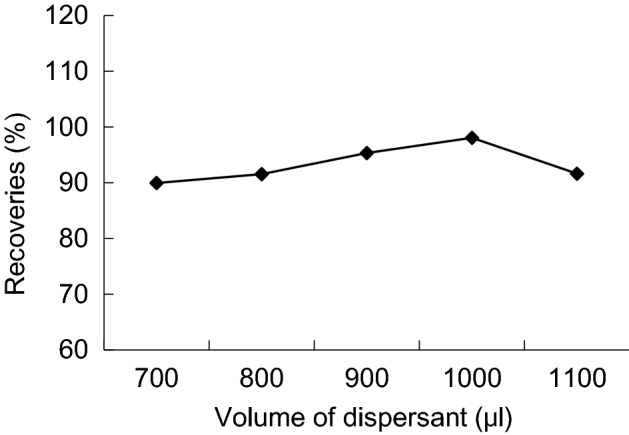
Recoveries of different volume of dispersant.

Experiment with different volume (700, 800, 900 and 1000 μL) of acetone and 22 μL trichloroethane as extraction system, after extraction of centrifugal, sample injection 1 μL sedimentation volume, of the peak area increased with the increase of dispersing agent volume. The result is due to the volume of acetone increased, makes a certain amount of trichloroethane more dispersed in water, the the extraction efficiency relative higher. When acetone volume is 1000 μL, peak area reached the highest. So select volume of acetone is 1000 μL (Figs. 8, 9).

### Effect of extraction time and centrifugal time

**Figure 10:**
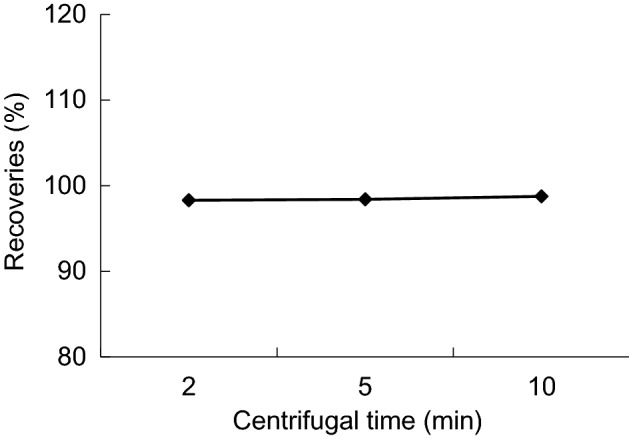
Recoveries of different centrifugal time.

For this study, chose 3500 r/min, and the centrifugal time is 2, 5, and 10 min, with the increase of the centrifugal time, the peak area of chlorpyrifos keep the same level, centrifugal time had no significant effect on the extraction efficiency. extraction agent of scattered in the mixture as long as through the short time of centrifugal can deposit to the bottom of the tube, it is one of the great advantages in this DLLME method, so choose 2 min is more timesaving (Fig. 10).

### Effect of concentration of salt

**Figure 11:**
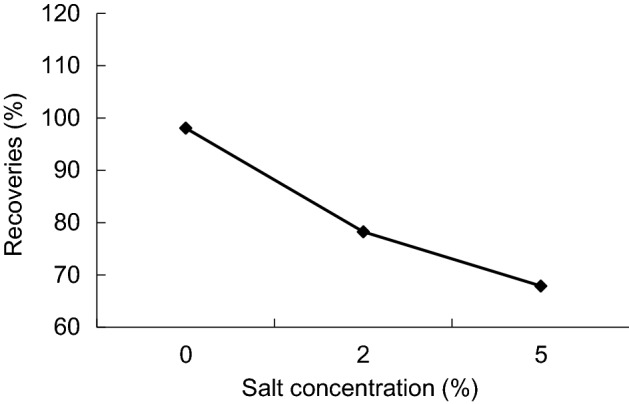
Recoveries of different salinity.

In this experiment, add NaCl change salt concentration in the water phase, concentration of 0%, 2% and 5%. Results (Fig. 11), a mixture of the increase of the extraction solvent solubility in the aqueous phase with the salt concentration increased, but volume of precipitated phase increased in the end, affecting the extraction efficiency of method, so the experiment without salt.

### Method validation

Th linear range of chlorpyrifos was 0.1–10 mg/L. Results show, in the optimized conditions, the peak area of chlorpyrifos had good linear relationship within a certain range, the Regression equation was Y = 16086x − 1448, the regression coefficients were greater than 0.999. The LOD was 0.02 μg/mL. The enrichment factor was 554.51. The recoveries for standard addition was 98.07%, the intra-day relative standard deviations (RSD, n = 4) was 4.61%, the veracity and accuracy of the method can meet the requirement of pesticide residue analysis (Table 1).

**Table 1:** Recovery assay, Recovery assay, precision (repeatability) and trueness of target compounds in greentea.

	Spiked sample					RSD (%) (N = 4)
	1	2	3	4	Average	
Volume (μL)	9.50	8.20	8.50	9.40	8.90	6.31
Sampling concentration (ng/mL)	9.68	12.77	11.51	10.41	11.09	10.52
Enrichment factor	483.90	638.39	575.25	520.51	554.51	10.52
Recovery (%)	91.94	104.70	97.79	97.86	98.07	4.61

## Discussion

In the DLLME method, extracting agent is one of the important factors affecting the extraction efficiency, then the main principle of choose it is: the extraction ability of extracting agent on the target have higher; Density is greater than the water and insoluble in water; To target without interference, the qualitative and quantitative analysis of the target will not affected.

Dispersing agent should be able to dissolved extraction agent completely, and soluble in water. Effect of dispersant is maximize for extraction agent with the contact area of the sample solution, its solubility in water, the greater the formation of droplets will be smaller, and the bigger with the target contact area, and the extraction efficiency higher. Volume of dispersant will affect the dispersion degree of extraction agent in water, which affects the sedimentation volume, which influence the extraction efficiency.

The extraction time in this method, refers to after the dispersant, extraction agent and ultrapure water mixture to before the centrifugal a period of time. This experiment select extraction time is 1, 3, and 5 min. The results showed extraction time had no significant effect on the extraction efficiency, and extraction time 1 min enough to form emulsion to the water phase of target transfer to the organic phase to two phase equilibrium.

Salt effect is often in the process of microextraction evaluation of a parameter, the change will change the concentration of salt in the water solution of ionic strength, is different of the extraction effect. The increase of ionic strength makes increase solubility of extractant in the aqueous phase, to improve recovery, but increase the volum of the sedimentary facies after the centrifugal, the concentration decreases of the target in sedimentary facies, enrichment coefficient dropped significantly.

### Comparison of the method with other approaches

The analytical characteristics (Recovery, EF, RSD, and LOD) of the method and other previously published approaches for the analysis of the target analytes in different samples are compared and summarized in Table 2. The repeatability of the method is good and the RSDs are comparable or better than the others. The Recoverys of the method are comparable or better than those of the other mentioned methods. As it is seen from the results, in comparison with other approaches, this method shows very high EFs. In addition, compared with other methods, this method also has the advantages of less solvent consumption, less pretreatment time, and less sample requirement.

**Table 2:** Comparison of the presented method with the other methods used in preconcentration and determination of the studied pesticides.

Sample	Pesticide	LOD (ng/mL)	EF	RSD (%)	Recovery (%)	Method	Ref.
Tea	Fenpropathrin	0.3	–	12.4	86.9–98.3	DSDME–GC–ECD^a^	^54^
Fruit juices	Chlorpyrifos	2.89	–	7.3	73–82	MWCNTs–SPE–GC–NPD^b^	^55^
Fruit juices	Chlorpyrifos	0.63	714	4.8	87–94	PCL–g–GQDs–based DSPE–DLLME–GC–FID^c^	^56^
Fruit juices	Chlorpyrifos	0.98	663	5.2	86–99	DSPE–DLLME–GC–FID^d^	^57^
Water	Haloxyfop-R-methyl	4.35	171	3.12	78.4	SDLLME–HPLC–UV^e^	^58^
Wine	Oxadiazon	0.1	–	13.5	–	HS–SPME–GC–MS^f^	^59^
Different drinks and liquids	Phthalate esters and antioxidants	0.67–1.24	205–235	3.8–5.7	80–115	DSPE–DLLME–GC–FID^g^	^60^
Tea	Chlorpyrifos	20	554.51	4.61	98.07	DLLME-GC-FPD^h^	This method

^a^Directly suspended droplet microextraction–gas chromatography–electron capture detector.

^b^Multi-walled carbon nanotubes–solid phase extraction–gas chromatography–nitrogen phosphorus etection.

^c^Poly (ε-caprolactone) grafted graphene quantum dots–based dispersive solid phase extraction–dispersive liquid–liquid microextraction–gas chromatography–flame ionization detection.

^d^Dispersive solid phase extraction–dispersive liquid–liquid microextraction–gas chromatography–flame ionization detection.

^e^Sequential dispersive liquid–liquid microextraction–high performance liquid chromatography–ultraviolet detector.

^f^Headspace–solid phase microextraction–gas chromatography–mass spectrometry.

^g^Dispersive solid phase extraction–dispersive liquid–liquid microextraction–gas chromatography–flame ionization detection.

^h^Dispersive liquid–liquid microextraction–gas chromatography–flame ionization detection.

## Conclusions

In the present work, a simplified and rapid sample pretreatment procedure based on DLLME was introduced as an efficient method for the extraction of chlorpyrifos from green tea sample before their determination by GC-FPD. In this research, for the first time, the acetone was used not only as solvent to extract but also dispersant, trichloroethane was used as the extractant. the flame photometric detector is highly selective to organophosphorus compounds, so the organic solvent can directly into the chromatographic instrument analysis after DLLME operation, the interference of the impurity on the analysis is quite small, and a better extraction effect was obtained under the optimized extraction condition. As the outcomes indicate, the method has some remarkable characteristics such as high EF (554.51), low RSD (4.61%), and good Recovery (98.07%). These characteristics reveal that the established analytical approach can be reliably used for the determination of trace levels of chlorpyrifos in green tea sample, and all kinds of analysis parameters meet the requirements of the pesticide residues analysis for agricultural products and the sensitivity, it to broaden the application range of the DLLME techniques also is of great significance.
